# Broadband S-Parameter-Based Characterization of Multilayer Ceramic Capacitors Submitted to Mechanical Stress Through Bending Tests on a PCB

**DOI:** 10.3390/mi15111386

**Published:** 2024-11-16

**Authors:** Victoria Gutiérrez-Vicente, Jesús Alejandro Torres-Torres, Reydezel Torres-Torres

**Affiliations:** 1 Instituto Nacional de Astrofísica, Óptica y Electrónica (INAOE), San Andrés Cholula 72840, Mexico; reydezel@inaoep.mx; 2 Escuela de Laudería, Instituto Nacional de Bellas Artes y Literatura (INBAL), Queretaro 76000, Mexico; lau.jesustorres@inba.edu.mx

**Keywords:** MLCC, S-parameters measurements, PCB, bending test

## Abstract

A full characterization of multilayer ceramic capacitors including variations in capacitance, series resistance, and series inductance is accomplished by measuring their RF response while being submitted to mechanical stress. This allows for the first time quantifying the degradation of the device’s RF performance when cracks form within its structure. In this regard, the main challenge is designing an interface for measuring the high-frequency response of a capacitor using a vector network analyzer as a bending test on a PCB in progress, which is achieved here by using a microstrip-based test fixture. The results indicate that there is an overestimation of its response to microwave stimuli when considering only the degradation impact as a reduction in capacitance. Capacitors of representative sizes and capacitances are analyzed to show the usefulness of the proposal, whereas the validity of the results is verified by observing the correlation with measurements collected using microprobes and performing optical inspections of cross-sectioned samples.

## 1. Introduction

Multilayer ceramic capacitors (MLCCs) are key components in high-speed electronics, serving as coupling or decoupling elements [1,2], and mitigating voltage fluctuations in power distribution networks (PDNs) [3]. Nevertheless, the useful bandwidth of MLCCs is limited by their inherent parasitic effects, represented by means of equivalent lumped elements: a series resistance (R_MLCC_) and a series inductance (L_MLCC_) [4]. Hence, circuit designers require precise knowledge of these elements during system development stages and when performing reliability analyses. This information allows for assessing the suitability of a given MLCC for a particular application.

Bear in mind that to accomplish the miniaturization demands of electronic circuits, MLCCs are built using dielectric materials with high dielectric permittivity. Unfortunately, these materials exhibit properties that make the devices prone to suffering fracture when mounted on a PCB. This is of relevance mainly for MLCCs belonging to the Class II category [5], which may experience significant and even critical damage due to PCB warpage [6]. In this regard, the bending of a printed circuit board (PCB) transmits tensile forces to the capacitor through the solder fillets and generates cracks that pass through its internal body [7]. These cracks significantly impact the electrical characteristics of the device as well. In fact, research teams have conducted work in this direction, but solely focusing on the capacitance variation induced by the device degradation during bending tests [8]. Nevertheless, to the extent of the authors’ knowledge, there are no reports within the open literature about the corresponding impact on the series parasitics. This omission arises from the need for high-frequency electrical measurements to determine R_MLCC_ and L_MLCC_ [9], which becomes challenging when the device is subjected to mechanical stimuli. Take into consideration that the test fixture must remain mechanically stable and maintain a consistent electrical response during the tests.

Regarding the high-frequency characterization of devices, vector network analyzers (VNAs) are the preferred choice due to their wide availability in research laboratories. In this case, however, it has been pointed out that test fixtures introducing minimized parasitics are necessary to reduce the uncertainty in the characterization process [10]. This can be achieved, for instance, employing coplanar microprobes [11]. Unfortunately, this option does not offer mechanical robustness during a bending test since maintaining constant probe-to-pad contact is not guaranteed. Alternatively, it is possible to obtain acceptable results by performing a two-port measurement where the MLCC is configured in a shunt connection [9], provided that the appropriate de-embedding of the test-fixture effects is performed [4].

Here, we present the full characterization of MLCCs while enduring degradation due to flex cracking. For this purpose, a bending test is designed based on a two-port microstrip line fixture on a PCB, which allows for inducing cracks on an MLCC and collecting *S*-parameters to monitor the gradual electrical degradation of the device. Moreover, the methodology enables the determination of the breakdown strain as well as the capacitance, series inductance, and resistance of MLCCs under mechanical stimuli. Thus, when applying the proposal, MLCCs with nominal capacitances of 0.1 μF and 1 μF for three standard sizes are characterized from 40 kHz to 6 GHz.

## 2. Description of Prototypes

The analysis presented in this paper is based on measuring the microwave electrical response of MLCCs using a VNA, before and during the gradual application of a bending stress. Hence, an analysis can be performed on the device’s performance degradation when soldered onto a board that undergoes deformation. This section is dedicated to describing the employed devices under test (DUTs), the measurement interface, as well as the electrical measurements including the corresponding de-embedding of the test fixture.

### 2.1. MLCCs Under Analysis

**Figure 1:**
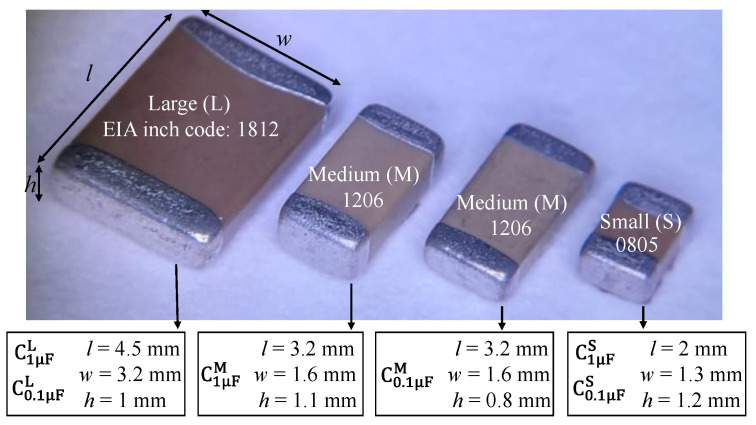
Photograph illustrating the sizes of the analyzed MLCCs. The superscript in the device names indicates the size: large (L), medium (M), and small (S). The subscript indicates the nominal capacitance.

There is a wide variety of package sizes for MLCCs [12]. Nevertheless, in applications where their microwave performance is relevant, it is desirable to use the ones exhibiting more compact size to reduce the associated parasitics [13]. Moreover, the required magnitude of capacitance in these cases ranges from a fraction of a microfarad to some microfarads. Figure 1 shows MLCCs of standard sizes that are typically analyzed in the literature since they allow for fulfilling these requirements [5]. In this figure, the code given by the Electronic Industries Association (EIA) specifying the package dimensions is indicated. Here, several capacitors of these particular sizes and two capacitance magnitudes (i.e., 0.1 μF and 1 μF) are used during the experiments, and are referred to as large (L), medium (M), and small (S). Thus, for instance, in Figure 1, the symbol $C_{1\mu F}^{L}$ refers to a MLCC with a package identified by the EIA code ‘1812’ and a nominal capacitance of 1 μF. It is important to remark that all the analyzed capacitors belong to Class-II are made of X7R dielectric material, which are of common use in practice for achieving relatively large capacitance in a compact space.

### 2.2. PCB Design

**Figure 2:**
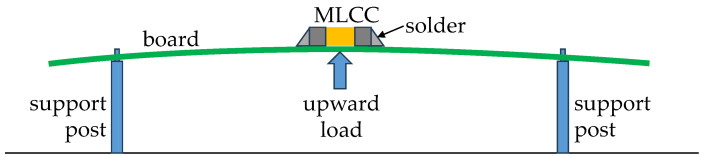
Conceptual illustration (not to scale) of the bending test.

To emulate a scenario where the MLCC is subjected to mechanical stress, the experiment depicted in Figure 2 is commonly used [14,15]. Notice that the device is soldered to a PCB held by supports, and the bending force is applied upwards below the MLCC. In this regard, the board is made of standard FR-4 material with a thickness of 1.5 mm, and it has nominal relative permittivity and loss tangent values of 4.4 and 0.02, respectively. The suitability of this board is based on the fact that the series inductance is on the order of tenths of nanohenries, making the corresponding effects noticeable at frequencies starting from the megahertz range and clearly observable at a few gigahertz. In this frequency range, FR-4 materials remain adequate for building prototypes for microwave characterization purposes [16]. Here, the upper limit for device characterization to encompass the range where the series inductance can be determined from experiments is 6 GHz.

In this work, it is necessary to measure the electrical response of the MLCC while the board is being bent. If only capacitance measurements are required, a multimeter can be connected by soldering cables to the terminals of the MLCC [17]. In this regard, limiting the experimentation to capacitance alone may be sufficient if the goal is to conduct detailed analyses of the effects of non-ideality on the properties of the constituent materials, such as the variation in permittivity of the dielectric material between the plates or the occurrence of flexoelectric polarization [18]. These aspects fall outside the scope of this work, which focuses more on analyzing the device’s response at microwave frequencies, where the reactive capacitance of the device is very low and not particularly sensitive to these effects.

**Figure 3:**
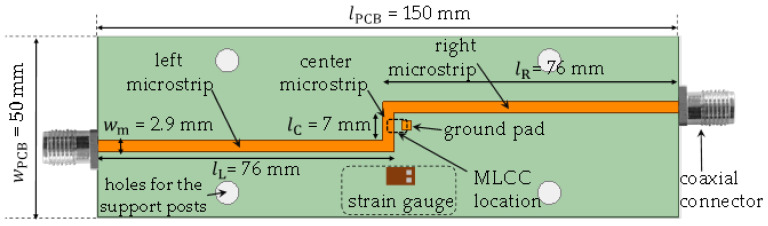
Sketch showing the details of the designed PCB for mounting the MLCCs.

Notwithstanding, either coaxial or probe interfaces are necessary to collect the S-parameters of the device for microwave response characterization. This presents the additional challenge of maintaining the electrical characteristics of these interfaces while applying mechanical stimuli. Consequently, due to their mechanical robustness, coaxial connectors are selected to interconnect the prototype with the VNA. However, it is important to place these connectors away from the point where the upward load is applied to avoid any changes in the connector interface. For this reason, the interconnection between the connectors and the DUT is achieved using microstrip lines. The proposed test fixture is shown in Figure 3.

To ease the bending of the MLCC, Figure 3 illustrates that the board is made longer than it is wide, and the device’s terminals are positioned along the length of the board. With this arrangement, a gradual formation of cracks can be achieved within the internal structure of the MLCC. Now, to achieve the interconnections required for the electrical measurements, the coaxial connectors are located at the edges of the board. Here, consider that due to the significant parasitic effects introduced by these interconnects, it is strongly recommended to measure MLCCs in a two-port shunt configuration to reduce uncertainty in the electrical characterization process [19]. Thus, for the implemented prototype, the three-section microstrip line, also depicted in Figure 3, is used. The target characteristic impedance of these lines is 50 Ω; therefore, considering the PCB characteristics previously described, the corresponding width is 2.9 mm. This is confirmed by measuring a straight microstrip line of 9 cm, which also verifies that the return loss at each port, including the connectors, is below –20 dB, while the insertion loss is less than 0.25 dB/cm. This indicates the appropriateness of these lines for the purposes of this work.

To complete the description of the test fixture with the aid of Figure 3, note that one of the MLCC terminals is directly soldered to the center microstrip line, while the other is connected to ground through a pad linked to the bottom plane using a vertical via [4]. Additionally, it is important to mention that a strain gauge is mounted on top of the board and parallel to the MLCC, allowing for the quantification of the strain experienced by the board under specific mechanical stimuli. This gauge is connected to a Wheatstone bridge, which produces an output voltage from which the strain can be straightforwardly computed [20].

## 3. Electrical Characterization of the MLCC

For characterizing the MLCC from *S*-parameter measurements, a model is required to represent the test fixture. Based on this model, a de-embedding procedure can be used to obtain the DUT response without the influence of the connectors, microstrip lines, and other parasitic effects. This section presents the details regarding the determination of the MLCC’s electrical characteristics.

### 3.1. Coaxial S-Parameter Measurements

**Figure 4:**
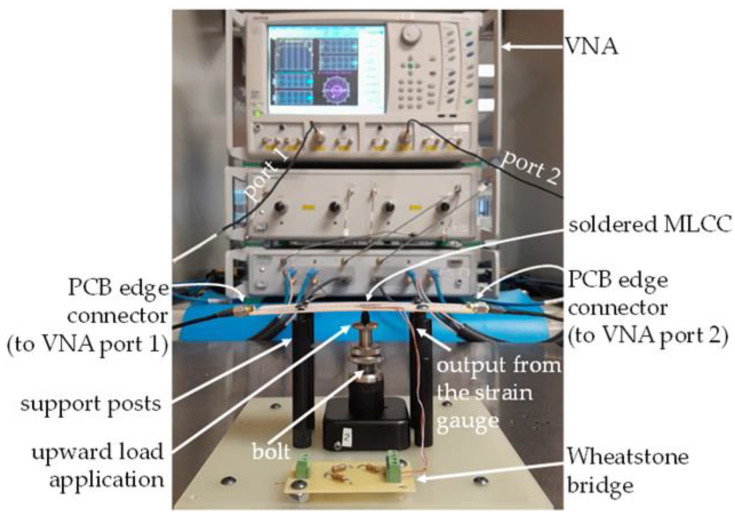
Experimental setup for measuring the S-parameters while gradually applying mechanical stimuli. The voltage source and multimeter connected to the Wheatstone bridge are not shown.

**Figure 5:**
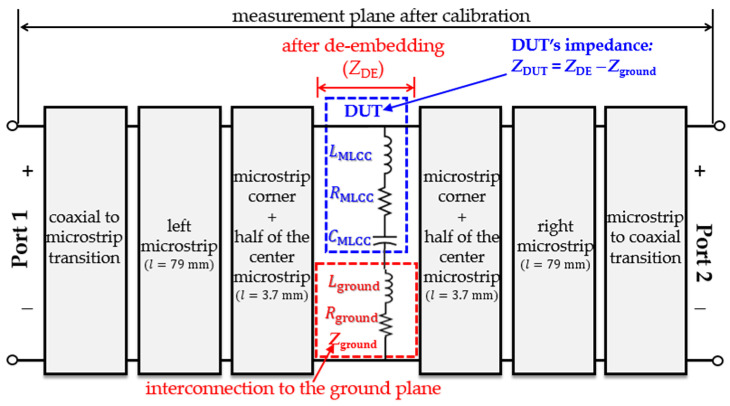
Two-port network model representing the interconnects used to access the MLCC.

With the purpose of obtaining the electrical response of the MLCCs, S-parameters are measured for the test fixture shown in Figure 3. These measurements are performed from 40 kHz to 6 GHz considering a logarithmic frequency sweep that allows for inspecting both the low- and high-frequency response of the device. Figure 4 shows the configuration of the VNA setup, which is previously calibrated using short-open-load-through (SOLT) mechanical standards and the corresponding mathematical algorithm. After calibration, the measurement plane is shifted towards the end of the coaxial cables at their interface with the sub-miniature type-A (SMA) connectors attached to the prototype. In addition, this procedure establishes a reference impedance Z_ref_ = 50 Ω for the collected S-parameters. Once the setup is calibrated, the DUT remains embedded within the test fixture, as illustrated by the cascaded block model in Figure 5. Thus, the effects of the connectors and the microstrip lines feeding the DUT are eliminated by applying a two-tier de-embedding process [16], which utilizes the measurements of two straight lines differing in length but presenting the same cross-section and coaxial terminations as the line shown in Figure 3. During this process, the effects of the microstrip corners are also taken into account to further enhance the de-embedding, using a circuit model in the Keysight ADS simulator.

After de-embedding, the reference plane of the *S*-parameters is shifted near the DUT. These *S*-parameters are then transformed to *Z*-parameters using the corresponding two-port network conversion, which enables the determination of the impedance referred to as *Z*_DE_ in Figure 5. Bear in mind, however, that this impedance still includes the parasitic effects represented by the impedance *Z*_ground_, which is associated with the interconnection that brings one of the MLCC’s terminals to ground. In order to determine the series resistance and inductance that allow for modeling *Z*_ground_, an additional dummy structure consisting of a short-circuited pad was also measured. Consequently, the impedance associated with the DUT was calculated as:

(1) $$Z_{DUT}=Z_{DE}-Z_{ground}$$

### 3.2. Probe S-Parameter Measurements

**Figure 6:**
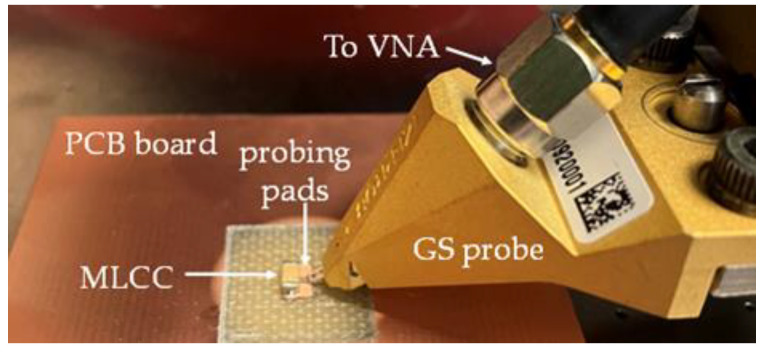
Photograph illustrating probe measurements performed.

Before starting to measure the MLCCs using the previously described test fixture, it is necessary to corroborate that the de-embedded *S*-parameters from the coaxial measurements accurately represent the electrical response of the device alone. For this purpose, an additional set of measurements with minimized parasitics are performed for MLCCs of the different considered sizes. These measurements are collected using a ground-signal (GS) coplanar probe with a 1000 µm pitch, which allows obtaining one-port reflection coefficient (Γ) data for the MLCCs within the same frequency range as for the coaxial case. The details of this experiment are shown in Figure 6. In this case, a short-open-load (SOL) calibration was applied using an impedance standard substrate (ISS) that shifts the reference plane to the end of the probe tips and sets *Z*_ref_ = 50 Ω. Furthermore, the effect of the GS-configured pads where the MLCCs are soldered is de-embedded using a simple procedure involving the separate measurements of the pads in open and short-circuited conditions. Hence, from the corrected reflection coefficient data, the impedance of an MLCC can be calculated using the following Equation (2):

(2) $$Z_{DUT}=Z_{ref}\frac{1+\Gamma }{1-\Gamma }$$

**Figure 7:**
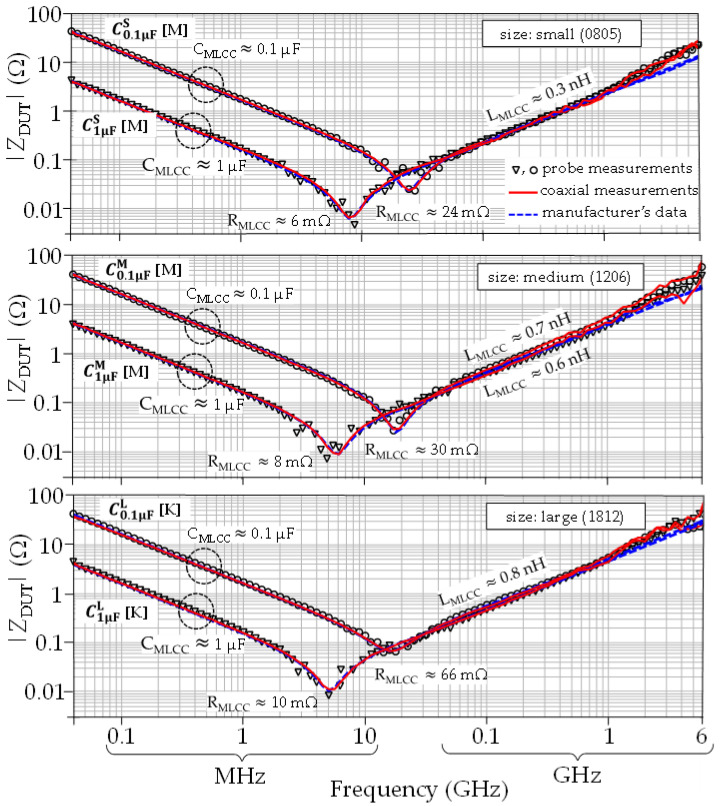
Comparison of impedance curves obtained from probe and coaxial measurements for the three MLCC sizes and two capacitance magnitudes considered in this work. The curves show agreement with the data provided by the manufacturer [21,22], which are also included in the plots.

Figure 7 shows that the curves obtained from coaxial measurements (i.e., Equation (1)) and probe measurements (i.e., Equation (2)) exhibit no noticeable difference within the measured frequency range for the three MLCC sizes and the two capacitance magnitudes considered in this work. This verifies the validity of the de-embedding procedure performed for the coaxial measurements and the feasibility of accurately determining the MLCC response from the proposed test fixture. For completeness, the data provided by the device manufacturer are also included in Figure 7, showing agreement with our measurements.

### 3.3. Application of Strain

The basic idea of the experiments performed here is firstly obtaining the *S*-parameters of an MLCC embedded within the coaxial test fixture shown in Figure 4 without applying any mechanical stimulus. Then, the upward load indicated in this figure is exerted beneath the board under the capacitor by using a bolt with adjustable height. This height is increased in steps of 300 μm, which in turn results in a gradual increase of approximately 200 μ-strains (i.e., strains × 10^−6^) on the board. Bear in mind that to accurately determine the actual stimulus applied each time, the strain gauge output is monitored at every step through the Wheatstone bridge.

Once a height step is settled, the board is left to rest for one minute, and then the *S*-parameters are measured again. At this point, it is important to highlight that it was previously verified that the electrical response of the test fixture alone was not modified within the considered range of bending strain; thus, any change in the *S*-parameters of the MLCC measured through this structure corresponds to changes in the response of the device itself.

After completing the measurement, *Z*_DUT_ is obtained from (1), which enables the determination of the MLCC’s equivalent circuit elements (i.e., C_MLCC_, L_MLCC_, and R_MLCC_ in Figure 5) from a log–log plot like those shown in Figure 7. Subsequently, the bolt height is increased, and the *S*-parameters are re-measured. The procedure continues until *C*_MLCC_ exhibits a reduction higher than 90% of its initial magnitude.

**Figure 8:**
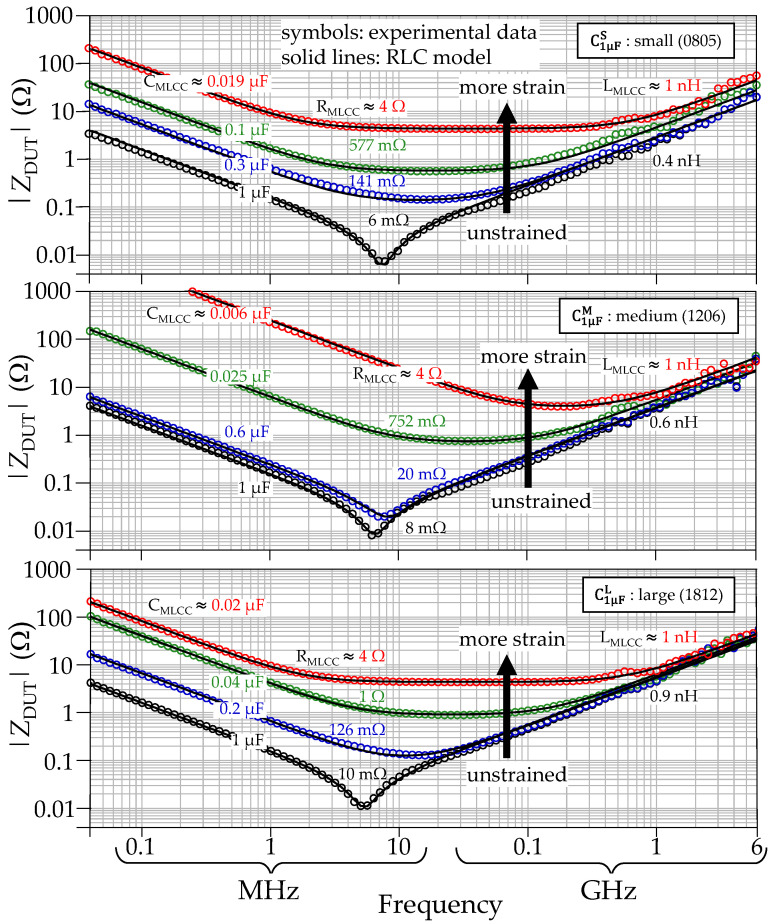
Impedance curves for MLCCs with nominal capacitance of 1 μF under different strain stimuli. The plots, from top to bottom, correspond to one small-, one medium-, and one large-size device.

**Figure 9:**
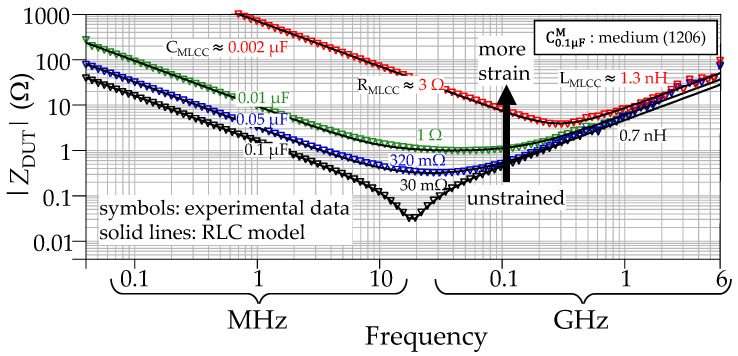
Impedance curves for medium-size MLCC with nominal capacitance of 0.1 µF.

The experiments were conducted for four MLCCs of each one of the three considered sizes and the two capacitance magnitudes, totaling 24 characterized devices. Figure 8 shows the results for three of these capacitors exhibiting the same nominal capacitance of 1 μF and the different considered sizes. These plots correspond with representative strain levels to avoid overcrowding and include those obtained when the device is unstrained and when the capacitance has been reduced by about 90%. For illustrative purposes, also the curves for one of the medium-size MLCCs with nominal capacitance of 0.1 μF are shown in Figure 9. Notice that, as well as for the cases shown in Figure 8, there is a gradual change in the capacitance between the unstrained condition and the final curve, corresponding to a reduction beyond 90%. Interestingly, the resistance R_MLCC_ is the parameter that suffers more change by increasing from some tens of milliohms to a few ohms, whereas L_MLCC_ increases within the same order of magnitude. A deeper discussion about the results of these experiments is presented in the following section.

## 4. Results

**Figure 10:**
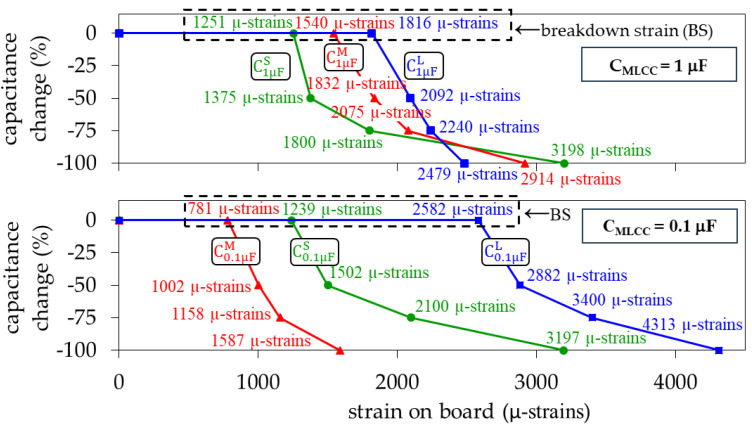
Change of capacitance versus applied strain for the considered sizes and capacitance magnitudes. Each data point was obtained from averaging measurements performed for four devices.

While gradually increasing the strain during the bending tests, the capacitance exhibited no variation until a limit was reached. This limit is referred to here as ‘breakdown strain’ (BS) and was determined from the last strain measurement at which no capacitance variation was detected in the measured *S*-parameters. Once the BS was exceeded, the capacitance started to drop as the strain increased. This is shown in Figure 10 for the two nominal capacitances and for the three MLCC sizes considered in this work. Since four MLCCs were measured for each case, every data point in these plots was obtained by averaging the measurements performed for these different devices.

Notice in Figure 10 that for the 1 μF MLCCs, the large device ($C_{1\mu F}^{L}$) is the one exhibiting higher BS, which is reduced for the medium size ($C_{1\mu F}^{M}$), whereas the small one ($C_{1\mu F}^{S}$) is the one presenting the lower BS. To explain this trend, consider that the tensile forces experienced by an on-board MLCC originate cracks that initiate at its bottom [23], close to the edge of the outer electrodes. Hence, the regions near the MLCC terminals are the most vulnerable to suffering mechanical damage. In a large MLCC, these regions are further from the point where the upward load is applied, thus requiring higher strain to suffer damage when compared with a smaller device. Nevertheless, it is worth noting in Figure 10 that for the 0.1 μF MLCCs, the medium-size MLCC ($C_{0.1\mu F}^{M}$) is the one presenting the lower BS. This comes from the fact that the height of this MLCC is smaller than that of the small one ($C_{0.1\mu F}^{S}$), which is shown in Figure 1. Therefore, our results point out that the stiffness of the capacitor, also associated with its height, is a determinant in the magnitude of BS. In addition, Figure 10 shows that once the MLCC is broken, the damage in the metal plates forming the inner electrodes yields a noticeable reduction in capacitance, which has been consistently reported in the literature [14,15].

**Figure 11:**
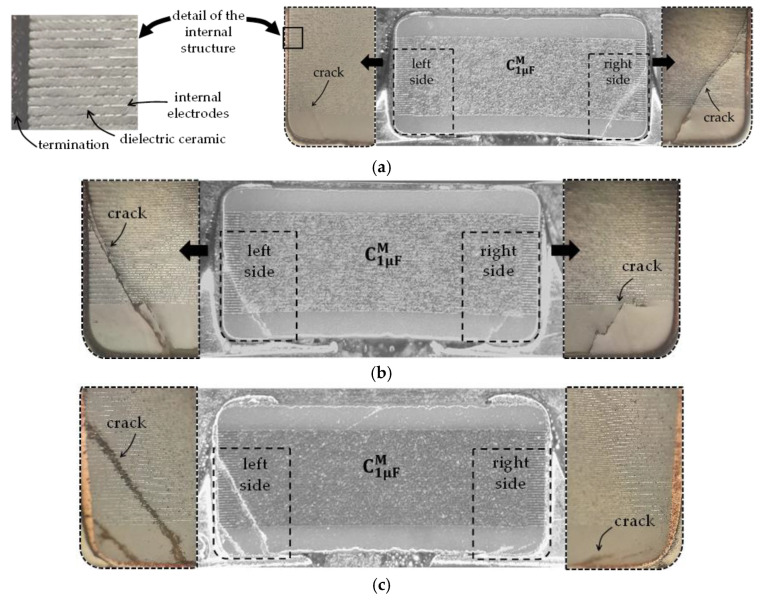
Photographs showing the cross-section of three different medium-size MLCCs with nominal capacitance of 1 µF, after monitoring a reduction of capacitance of approximately: (**a**) 30%, (**b**) 50%, and (**c**) 95%.

**Figure 12:**
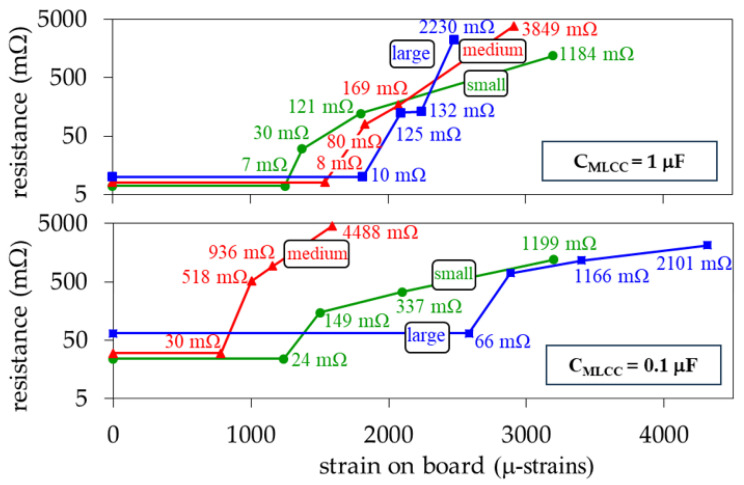
Average equivalent resistance (R_MLCC_) versus applied strain for the considered sizes and capacitance magnitudes.

Now, for inspecting the structural change of the MLCCs after breakdown, several devices were cross-sectioned after suffering a capacitance reduction of 30%, 50%, and 95% by following the sample preparation described in [24]. The corresponding photographs are shown in Figure 11, illustrating no evident variation in the cracks associated with the different degradation levels. This points out that once a crack occurs, applying more strain worsens the damage in the internal electrodes along the crack path. As a consequence, the capacitance is evidently reduced, but also the broken metal plates yield a substantial increase in the parasitic series resistance. In accordance with the experimental results obtained here, this increase is dramatic since the nominal resistance of an MLCC is typically within the order of a few milliohms, whereas it rises up to the range of ohms once cracks appear within the internal structure of the device. This is observed in Figure 12, which presents the resistance curves associated with the devices used to obtain the results in Figure 10.

**Figure 13:**
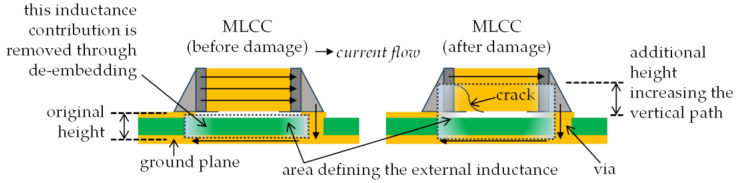
Conceptual illustration of the increase in the current path after a crack is formed, which yields a higher external inductance.

Regarding the equivalent series inductance, this parameter exhibits an increase as the bending test progresses. When the crack is formed, the current path is degraded. Specifically, as shown in Figure 11, since the bottom inner plates of the MLCC are prone to being damaged due to their proximity to the PCB solder points, the current flow is degraded at the lower levels. Hence, the alternating-current path is increased due to its confinement to the higher plates within the MLCC, as conceptually illustrated in Figure 13. Observe in this figure the depiction of the increase in the transverse area formed by the current loop after a crack is formed. This in turn yields an increase in the external inductance experienced by a signal propagating along the device and its corresponding return path through the ground plane. Bear in mind that applying Equation (1) removes the contribution of the PCB height to the area defining the external inductance; hence, the determined increase in inductance monitored through the de-embedded *S*-parameters is that associated solely with the MLCC.

**Figure 14:**
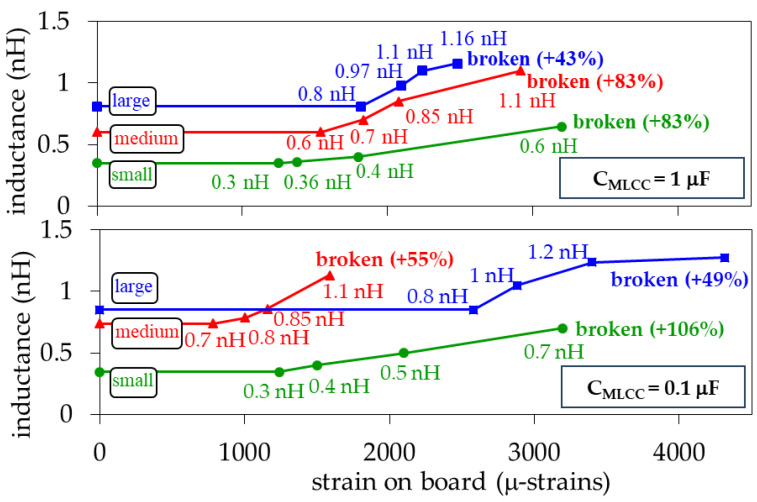
Average equivalent inductance (L_MLCC_) versus applied strain for the considered sizes and capacitance magnitudes.

Figure 14 shows the increase in inductance versus the applied strain for the analyzed capacitors. In all cases, the maximum increase occurred within one order of magnitude at the end of the strain experiments. In fact, for the worst case, when the capacitance was reduced to 10% of its original magnitude, the inductance approximately doubled the nominal value reported by the manufacturer. It is important to highlight that the form of the crack will determine the path followed by the current through the capacitor after damage; nevertheless, an interesting observation in the experimental data shown in Figure 14 is that the tallest capacitors, $C_{1\mu F}^{S}$ and $C_{0.1\mu F}^{S}$, which present *h* = 1.2 mm, are the ones exhibiting the higher inductance increase. This is attributed to a higher increase in the current path after damage, as illustrated in Figure 13.

## 5. Example of Application

**Figure 15:**
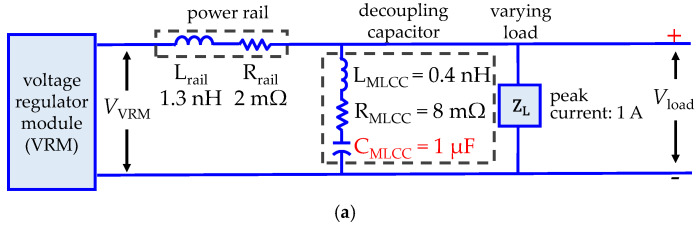

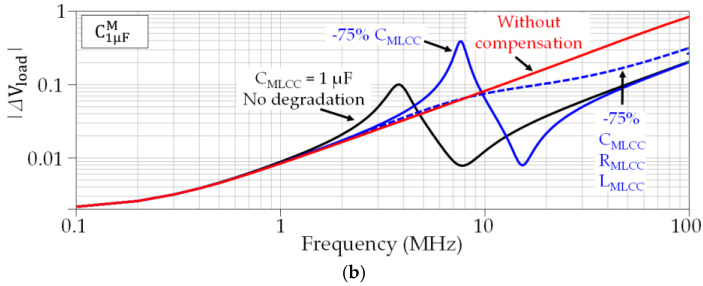
Simulations predicting the impact of the MLCC degradation when compensating a power delivery network (PDN): (**a**) simulated circuit and (**b**) magnitude of the AC voltage at the load considering only the capacitance reduction (solid line), and including also the variation of the parasitic components R_MLCC_ and L_MLCC_ (dashed line).

A common application of MLCCs is compensating the inductive effects occurring in power delivery networks (PDNs). In this case, the voltage fluctuations introduced by the inductance effects occurring throughout the power rail and the ground path are reduced by using MLCCs. Figure 15a shows the schematic of a simple PDN consisting of a power rail exhibiting typical parasitics, represented by L_RAIL_ and R_RAIL_ [25]. In this example, the electrical power is guided from a voltage regulator module (VRM) to a load demanding a time-varying current. Hence, to compensate for the voltage drop occurring at L_RAIL_, an MLCC with capacitance C_MLCC_ = 1 μF is used, which allows for maintaining the variation of the voltage delivered at the load ($\left|{∆V}_{load}\right|$) below 100 mV at frequencies up to 50 MHz. In fact, notice in Figure 15b that once the resonance region is surpassed, $\left|{∆V}_{load}\right|$ is well below the voltage curve obtained when the PDN is not compensated. Now, when the MLCC suffers a mechanical stress that yields a 75% reduction of capacitance, the simple model that assumes that the device’s parasitics are immune to the mechanical stress only predicts a change in the magnitude and frequency of resonance. Moreover, when considering this assumption, $\left|{∆V}_{load}\right|$ is predicted to be significantly lower than that obtained without compensation at frequencies above the resonance region. Conversely, when repeating the simulation considering the impact of the structural damage on the MLCC parasitics, the voltage curve is very similar to the one obtained without compensation. This result points out the fact that neglecting the impact of mechanical stress on the MLCC inductance and resistance may substantially overestimate the performance of a damaged device.

## 6. Conclusions

The electrical response of an MLCC can be measured at microwave frequencies while simultaneously performing mechanical stress tests by using a fixture based on microstrip lines on a PCB. It has been demonstrated that the accuracy in the obtained device response is similar to that obtained when directly measuring using microprobes. This enables the determination not only of the capacitance variations when the MLCC is degraded due to mechanical stimuli, but also the changes in the resistive and inductive parasitics, which require measurements at least starting at some tens of megahertz. Moreover, the electrical tests were complemented with optical inspections of the cross-sections corresponding to MLCCs exhibiting a different severity in the damage. From this additional analysis, it was observed that the interruption of the path for the AC current introduced by the cracks is the origin of the increase in the series resistance and inductance. In this regard, the stiffness of the capacitor is one of the important factors determining its robustness against crack formation, which in turn is dependent on the length, width, and height.

When using the proposed test fixture and methodology to characterize MLCCs, it is possible to predict the impact of their structural damage on the performance of practical circuits. In fact, it was demonstrated that neglecting the influence of mechanical damage on the series parasitics may lead to incorrect simulation results underestimating the negative effect of the MLCC electrical degradation.

## Data Availability

The original contributions presented in the study are included in the article, further inquiries can be directed to the corresponding author.
